# A new treatment for frostbite sequelae; Botulinum toxin

**DOI:** 10.1080/22423982.2016.1273677

**Published:** 2017-01-19

**Authors:** Arne Johan Norheim, James Mercer, Frauke Musial, Louis de Weerd

**Affiliations:** ^a^ The National Research Centre in Complementary and Alternative Medicine, NAFKAM, Department of Community Medicine, Faculty of Health Sciences, University of Tromsø - The Arctic University of Norway, Norway; ^b^ Institute of Military Epidemiology, Defense Medical Center, Norwegian Armed Forces, Sessvollmoen, Norway; ^c^ Medical Imaging Group, Institute for Clinical Medicine, UiT The Arctic University of Norway, Tromsø, Norway; ^d^ Department of Medical Physiology, Faculty of Medicine, University of Tromsø - The Arctic University of Norway, Norway; ^e^ Department of Radiology, University Hospital North Norway, Tromsø, Norway; ^f^ Department of Plastic Surgery and Hand Surgery, University Hospital North Norway, Tromsø, Norway

**Keywords:** Frostbite, cold injury, botulinum toxin, vasospastic disorder, thermography, non-freezing cold injury, quantitative sensory testing, angiography, military, army

## Abstract

Frostbite sequelae are a relevant occupational injury outcome for soldiers in arctic environments. A Caucasian male soldier suffered frostbite to both hands during a military winter exercise. He developed sensory-motor disturbances and cold hypersensitivity. Angiography and thermography revealed impaired blood flow while Quantitative Sensory Testing indicated impaired somato-sensory nerve function. Two years after the initial event, he received an off label treatment with Botulinum toxin distributed around the neurovascular bundles of each finger. After treatment, cold sensitivity was reduced while blood flow and somato-sensory nerve function improved. The successful treatment enabled the soldier to successfully pursue his career in the army.

## Introduction

Frostbite has for decades, been a relevant problem in the military, and continue to be so. In recent years, cold injuries have also become more prevalent within the civilian population due to increasing interest in extreme outdoor recreation activities. The increasing number of homeless people after the recent financial crisis constitutes another growing group of particularly vulnerable individuals [1]. At the same time, sufficient standard medical treatment is currently lacking [2].

Frostbite has been defined as injury to body tissues caused by exposure to extreme cold, typically affecting the extremities and often involving only the skin, which initially becomes white and hard, but in severe cases resulting in gangrene of deeper tissues and loss of the affected parts [3]. The pathophysiological process is caused by heat loss sufficient to cause ice crystal formation in superficial or deep tissue [1]. During the last two decades, newer therapies aimed at prevention of extensive tissue injuries have shown promising results in experimental studies and case reports [1,4,5]. In addition to the acute injury, frostbite is often associated with long-term sequelae [6]. These sequelae are less well studied and treatment is often difficult.

Depending on the type of cold injury, cold hypersensitivity, sensory loss, chronic pain, hyperhidrosis, growth plate disturbances and osteoarthritis may develop [7]. Long-term paraesthesia with occasional electric shock-type sensations have also been reported [8,9]. These sequelae may have a considerable negative impact on the quality of life.

For the military, these sequelae may compromise future operational capability of the soldier [10]. We present a case of a soldier who suffered from long-term sequelae from frostbite to his hands after a military exercise who was successfully treated with Botulinum toxin type A (*BTX*-A) injections.

## Case presentation

In April 2013, in Northern Norway, a 24 year-old Caucasian healthy male soldier participated in a qualifying ski march to become a combat officer in the Norwegian Defence Forces. He was wearing full winter protection gear including wind protectors over his gloves. Earlier that day the weather conditions were fine with an air temperature around −5°C and a light southerly breeze. During the ski march, he felt warm and took of his gloves. The weather deteriorated rapidly in the afternoon. Air temperatures dropped to around −15°C and a forceful wind was now blowing towards his right side. Although he noticed a chilling of his hands from the wind, he still felt subjectively warm. After approximately 90 minutes, he decided to put on his gloves again and noticed that the skin of his hands had turned a blue-grey colour. His fingers felt numb with loss of finger dexterity. Having put on his gloves, he continued marching. After 2 hours, he felt a burning pain in his hands and noticed that the fingers were swollen and that blisters had developed on his fingers, especially on the right hand side. Strongly motivated to pass the test, he did not seek medical attention and continued the march. His fingers became more painful and he felt loss of sensation. After the exercise, he was diagnosed with second-degree frostbite injuries to his hands and was treated conservatively. The blisters were covered with paraffin gauze and a dry bulky dressing was applied. The wound dressings were continued until the wounds had healed. Due to cold hypersensitivity and pain when exposed to even a mild cold challenge, he was assigned to indoor duties.

In 2014, he wanted to qualify for a higher rank in a combat group. The training for this takes place in Northern Norway with more than 6 months of winter during which temperatures may reach −40°C. In the autumn of 2014 he reported that his fingers still became rapidly cold, pale and totally numb as soon they were exposed to cold, even at a temperature of +5°C. He felt a constant pain and loss of sensation in his fingers resulting in reduced finger dexterity. He was deemed unfit for outdoor military service and had to leave the combat unit.

In May 2015, he was referred to the outpatient clinic for Plastic Surgery at the University Hospital North Norway. He was diagnosed with sequelae from frostbite. To exclude other pathological conditions further investigations were carried out. Angiography of the upper extremities showed open arteries on the lower arms, but all digital arteries were described as very thin with no dilatation after heat stimulation or hand/finger movements. No other pathology was found.

Dynamic Infrared Thermography (DIRT) [11] was performed to test the patient’s response to a cold challenge. This involved measuring the skin temperature of the dorsal aspect of both hands before, immediately after and for 3 minutes following a mild cold challenge (1 min. immersion of the hand in water at 20°C). During the immersion, each hand was covered by a thin plastic bag to avoid it becoming wet. The skin temperatures were measured with a high definition infrared camera. The patient was wearing outdoor winter clothing to stimulate peripheral vasodilation. Prior to the cold challenge, the patient reported that he felt warm. Additional obtained facial thermographic images showed the nose was vaso-dilated, presumably due to open arterio-venous anastomoses. DIRT showed a rapid onset of cooling and slow rewarming of the fingers after the cold challenge, similar to the slow warming subjects reported by Brandstrom [12]. However, in our patient we performed a mild cold challenge, resulting in a clear difference between the right and left hand, the right hand being cooler.

Quantitative Sensory Testing (QST) [13] indicated a loss of function with regard to non-painful stimuli in the mechanical and thermal domain (cold-, mechanical-, and vibration detection threshold). These findings corresponded well with the patients’ symptoms, namely loss of sensation in his fingers and reduced finger dexterity.

The soldier was interested in receiving treatment. He was informed on the off label use of *BTX*-A to treat vasospastic disorders and nociceptive/neuropathic pain disorders. He consented to a treatment with *BTX-*A. Each 100 unit vial of *BTX-*A (Allergan, Inc. Irvine, CA) was prepared in a standard manner with a concentration of 40 units/ml. The injections were administered at the neurovascular bundles in the palm of each hand at the level of the metacarpophalangeal joints (60 Units *BTX-*A per hand). This resulted in a dose of 12 units of *BTX-A* just at or proximal to the annular pulley A1 of the flexor tendon sheath of each finger.

At 3 weeks follow up the patient reported that he had less pain, warmer hands and improved sensory function, based on his own subjective evaluation. In November 2015, during the winter period, the patient reported improved cold tolerance and was able to perform normal outdoor activities. Angiography, DIRT and QST were repeated and the results were compared to the pre-treatment results.

**Figure 1.**
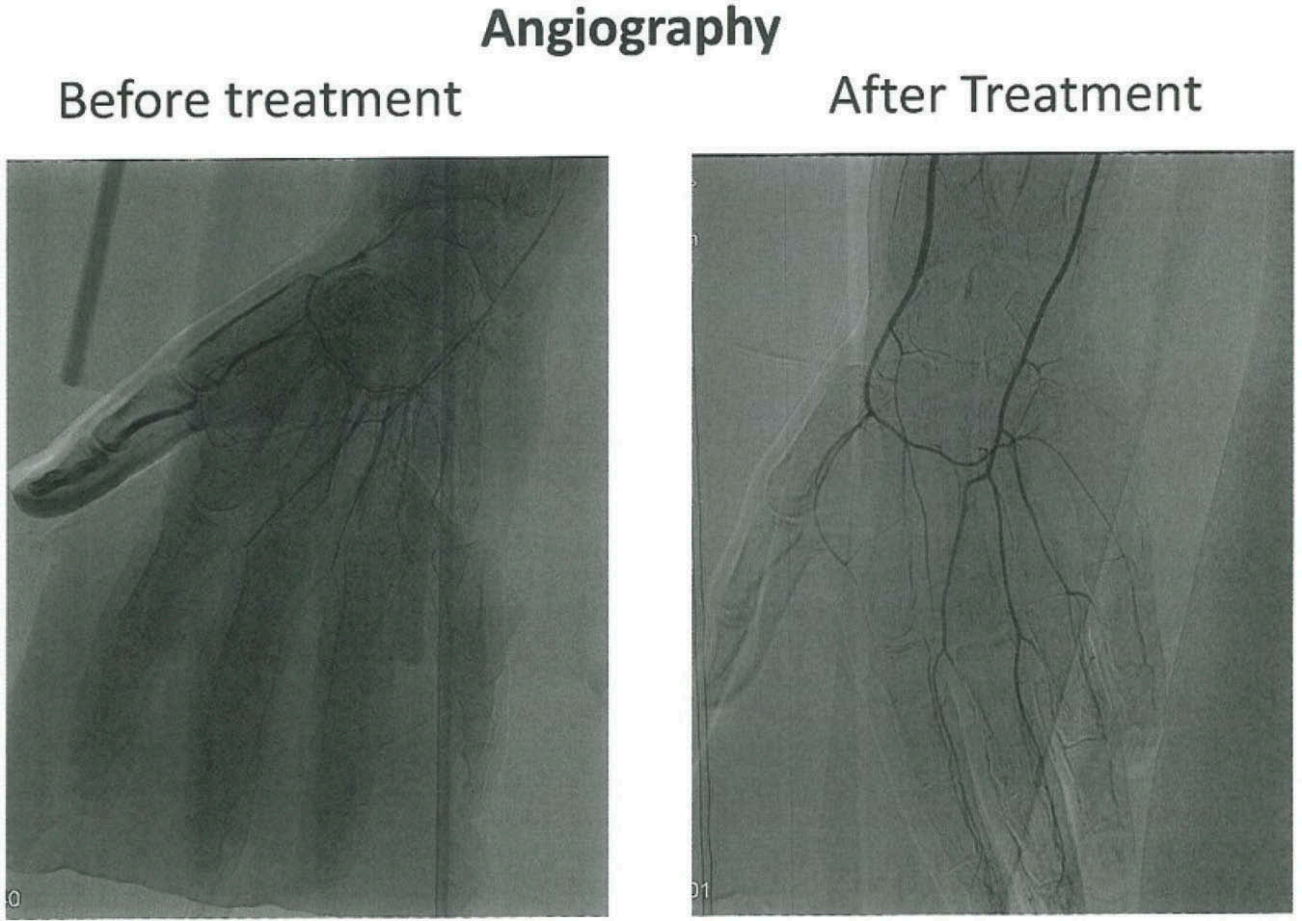
Angiography before and after treatment. Angiographic images showing the status of peripheral vessels in fingers. The pre-treatment picture show open arteries in the right forearm. The digital arteries in the hand are displayed extraordinary gracile. The blood-flow in the arteries are reduced, even if the fingers are stimulated with mild heat or undergoing exercise. The post-treatment images still show reduced peripheral vascularisation, however, the digital arteries are more vasodilated and less gracile compared with the pre-treatment display.

**Figure 2.**
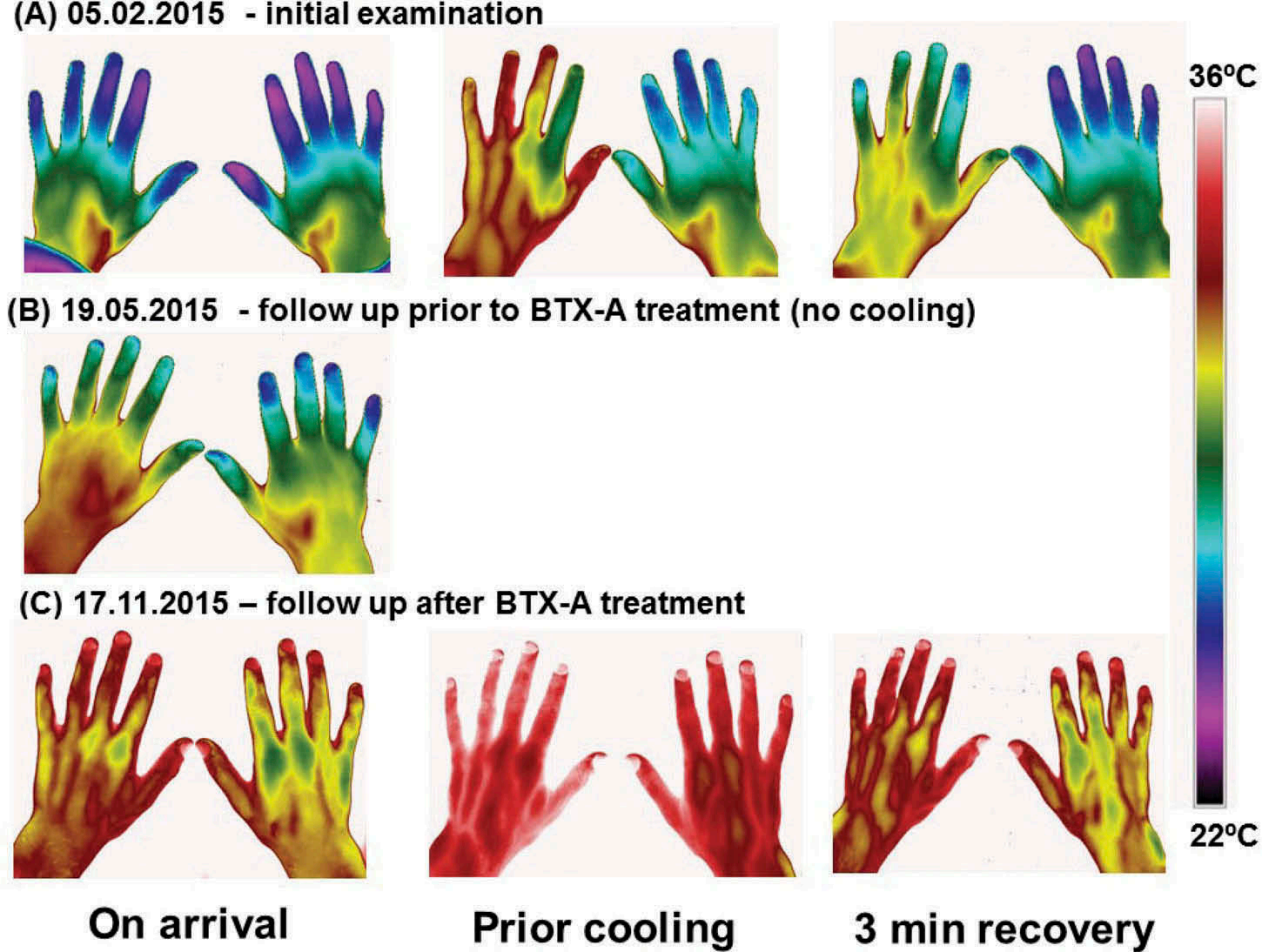
Thermography before and after treatment. Thermographic images of the hands taken on 3 different occasions. (A) the initial examination 05.02.2015; (B) follow up examination immediately prior to BTX-A treatment 19.05.2015 and (C) follow up examination after BTX-A treatment 17.11.2015. On the first and the third occasion, the patient’s hands was subjected to a mild cold provocation test (2-minute period of convective cooling using a desk top fan). The images were taken after the patient had had sat in the laboratory wearing warm clothes to ensure he was very mildly hyperthermic at the time of the examination. This was confirmed from the patient’s reported subjective feeling as well as from thermal facial images showing that the nose (open arterio-venous anastomoses) was vasodilated. The hand images were taken immediately after the patient arrived at the laboratory (on arrival), after the patient had been warmed up (prior cooling) and 3 minutes after the end of the cold provocation period (3 min recovery).

**Figure 3.**
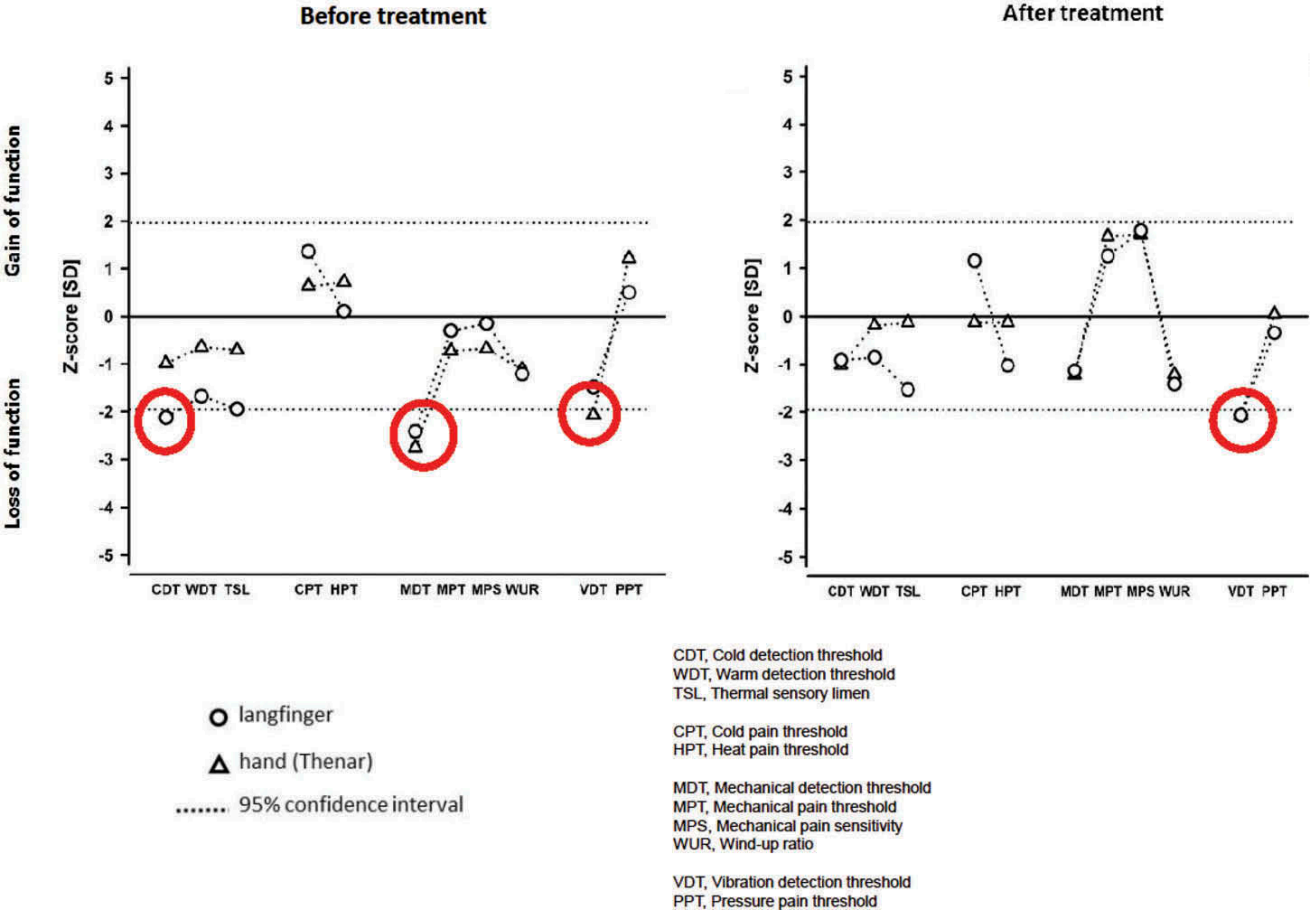
Quantitative Sensory Testing (QST) before and after treatment. The z-transformed pattern of 11 QST subtests (shown without dynamic mechanical allodynia (DMA) and paradoxical heat sensation (PHS), which were normal). QST reveals thermal hypoesthesia of cold stimuli (CDT) in the directly affected area (middle finger), most likely related to the loss of small fiber function (Aδ fibres). The ability to detect minimal mechanical stimuli (MDT) is reduced within (long finger), as well as outside (thenar of the same hand) the affected area, as is the ability to detect vibration (VDT) in the hand area. These mechanical deficits reflect most likely a loss of function of large diameter fibers (Aβ fibres). With the exception of VDT, treatment with BTX-A normalised the QST pattern. The red circles in the figure indicate values measured outside the two Standard Deviation (SD).

Angiography showed that the digital arteries were more dilated and more clearly visible (Figure 1). DIRT showed rapid and improved rewarming of all fingers compared to the pre-treatment examination although the rewarming of the right hand was still a bit slower compared to the left hand at all time points during the examinations (Figure 2). QST showed a normalisation of sensory function apart from a marginal reduction of the ability to detect vibration (Figure 3). Based on the encouraging results it was agreed to repeat the treatment. The same procedure with the same dose of BTX-A of 60 U (40U/per ml) per hand was used as during the first treatment.

At 6 weeks follow-up in January 2016, the patient reported even further improvement. The only side effect of both *BTX-*A treatments was a temporary weakness of the intrinsic muscles of each hand, the lumbrical and interosseous muscles to the 2–5 finger and the flexor pollicis breves, the opponents and adductor muscles of the thumb. This transitional weakness lasted about 3 weeks.

## Discussion

Frostbite can cause significant sequelae. A long-term study on 30 patients with significant frostbite sequelae revealed that 53% had cold hypersensitivity, 40% suffered from numbness and 33% had reduced sensitivity to touch [8,14,15]. Chronic pain is perhaps the most frequent complaint. These sequelae can cause considerable discomfort and functional limitations that may last for several years but can also have lifelong implications for the quality of life and professional career. The latter is of particular relevance for military personnel, since frostbite sequelae constitutes an occupational injury with a major career impact. Moreover, the increasing relevance among civilians is a matter of concern, in particular with regard to increasing interest in extreme winter sports activities [1,2] as well as in those with limited possibilities to protect themselves, such as homeless people.

The pathophysiology of cold injuries and sequelae remains still poorly understood although peripheral neurovascular damage plays an important role [6]. The treatment of these sequelae is difficult. Cold hypersensitivity and pain are clearly a threat to the functionality, such as in the present case of this soldier.

Recently, *BTX-*A has been successfully used in the treatment of Raynaud’s phenomenon, well known as an exaggerated digital vasospasm, most commonly due to stimuli like cold and emotional stress [15]. In these patients *BTX-*A improved pain control, decreased numbness and increased blood perfusion to the fingers. *BTX-*A blocks the release of the neurotransmitter acetylcholine at the motor end plate terminals and as a result, smooth muscle vasoconstriction is inhibited [16]. *BTX-*A has also two other effects. It blocks the transmission of norepinephrine and prevents sympathetic vasoconstriction of vascular smooth muscle [17]. In addition *BTX-*A blocks recruitment of specific α2- adrenoreceptors, which decreases the activity of chronically upregulated C-fiber nociceptors. This causes subsequent reduction in cold-induced vascular smooth muscle constriction and pain [18]. The above current knowledge lends weight to the idea of using *BTX-*A to treat frostbite sequelae.

Our study using angiography, DIRT and QST indicate that *BTX-*A has positive effects on skin perfusion, cold hypersensitivity and pain in a patient with frostbite sequelae. Further studies are warranted to investigate the long-term effects of *BTX-*A in these patients. Although this patient had received his injury some years prior to treatment, we speculate that early treatment of frostbite sequelae with *BTX-*A may be advantageous. Early treatment may reduce the symptoms associated with frostbite sequelae and thereby reduce the period in which the patient is unable to perform his or her normal duties as a soldier in a cold environment.
